# Stereotactic Body Radiotherapy for Treatment of Squamous Cell Carcinoma of the Tongue Associated with Human Papilloma Virus: A Case Report

**DOI:** 10.3389/fonc.2013.00126

**Published:** 2013-05-17

**Authors:** Brian Rodgers, Prakash Neupane, Chris Lominska, Yelizaveta Shnayder

**Affiliations:** ^1^Department of Otolaryngology-Head and Neck Surgery, University of Kansas Medical CenterKansas City, KS, USA; ^2^Department of Internal Medicine, Division of Hematology and Oncology, University of Kansas Medical CenterKansas City, KS, USA; ^3^Department of Radiation Oncology, University of Kansas Medical CenterKansas City, KS, USA

**Keywords:** HPV, SBRT, oropharyngeal cancer, recurrence, tongue neoplasms

## Abstract

Stereotactic body radiotherapy (SBRT) has emerged as a treatment for recurrent squamous cell carcinoma of the head and neck in the field of prior radiation. We report a case of its use in an human papilloma virus (HPV) positive patient with squamous cell carcinoma of the right base of tongue. The patient had complete response to treatment and modest toxicities were noted. This represents encouraging results that SBRT is also useful for salvage in patients with HPV positive disease.

## Introduction

Due to increasing rates of primary chemoradiation therapy, previously irradiated head and neck cancer patients with recurrent disease are being encountered more and more frequently (Ang and Sturgis, 2012). Stereotactic body radiotherapy (SBRT) has recently been successfully implemented as a treatment option for these patients.

Reports from case series cite encouraging statistics. Variable tumor response rates from 40 to 80% have been demonstrated. Toxicity estimates vary, with some series reporting low rates and others significant rates of major complications (Heron et al., 2009) (Cengiz). Consensus appears to be that SBRT is a viable option with good short-term control rates and modest acute toxicity due to increased precision and decreased duration of treatment (Heron et al., 2009; Roh et al., 2009; Siddiqui et al., 2009; Rwigema et al., 2011; Ang and Sturgis, 2012; Comet et al., 2012).

Human papilloma virus (HPV) is known to be a positive prognostic indicator in head and neck cancer. HPV positive squamous cell carcinomas of the head and neck are more amenable to treatment. Patients typically present with less substantial tobacco history. Five-year overall survival rates are above 80%. As a result, there is more emphasis on preventing treatment-induced morbidities such as xerostomia and dysphagia. Different treatment strategies may be useful in this population, as less aggressive treatment may be adequate and could help prevent chronic complications (Unger et al., 2010).

To our knowledge, the SBRT reirradiation literature to date does not address HPV status. We report an HPV positive patient treated with SBRT and discuss future areas of investigation.

## Case

FT initially presented to KUMC in 2009 with 20 pack-year smoking history and symptoms of dysphagia, odynophagia, and right neck mass for 2–3 months. He was 52 years of age and drank approximately 21 alcoholic drinks weekly. He did not chew tobacco or betel nut. Examination revealed a right level 2 neck mass and a right tongue base mass. Imaging including CT and PET along with endoscopy and biopsy lead to the diagnosis of T2N2aM0 squamous cell carcinoma. The base of tongue mass measured 2.6 cm in greatest dimension and the neck mass measured 1.9 cm × 2.5 cm. Polymerase chain reaction testing for pan HPV DNA as well as DNA for subtypes 6, 11, 16, 18, 31, and 33 was performed. Only pan HPV DNA was positive, while subtypes 6, 11, 16, 18, 31, and 33 were negative individually. Therapeutic options were discussed, including organ preserving concurrent chemotherapy/radiation or laser excision. Given coexisting cardiovascular comorbidities (aortic stenosis, hypertension) and possible compromise of voice and swallow, plan was made for chemotherapy and radiation.

The patient completed a regimen of two cycles of induction chemotherapy with Cisplatin and Docetaxel, followed by radiation therapy and concurrent cetuximab, which was completed in October 2009. Radiotherapy was delivered using a delayed concomitant boost technique with intensity modulated radiation therapy for parotid sparing. A total of 72 Gy was delivered to the primary and positive node. Approximately 1.8 Gy per fraction (54 Gy total) was delivered daily 5 days per week for a total of 30 treatments. Treatments were twice daily for the final two and a half weeks when a boost dose consisting of 12 fractions of 1.5 Gy (18 Gy total) was delivered in the afternoons, following the morning treatments of 1.8 Gy. The total time elapsed was 6 weeks. On restaging PET, he had hypermetabolic lymph nodes and underwent right modified radical lateral neck dissection (levels two through four) in December 2009, showing microscopic squamous cell carcinoma in 3 out of 15 nodes.

**Figure 1:**
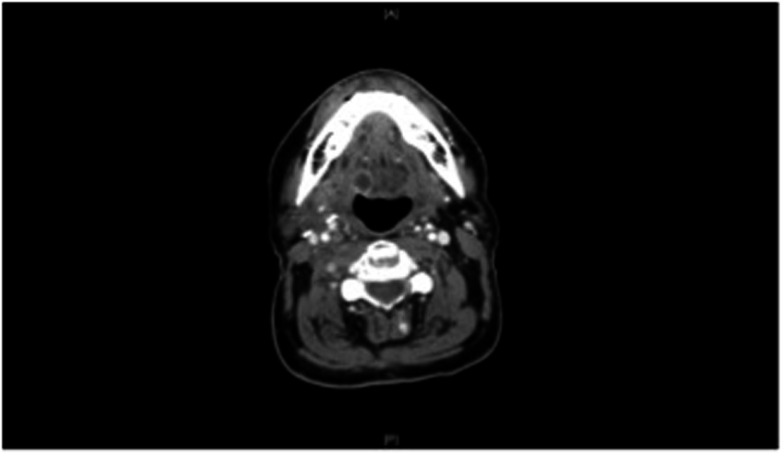
**CT neck of the neck with contrast, prior to treatment, demonstrates a submucosal irregularity that was ultimately biopsied and diagnosed as recurrent squamous cell carcinoma**.

Our patient did well over the next year, but unfortunately CT scan of the neck in September 2011 demonstrated an enlarged peripherally enhanced soft tissue lesion within the submucosa of the right tongue base measuring 1.3 cm × 1.5 cm × 1.1 cm, which was concerning for recurrent disease (Figure 1). PET scan confirmed a focus of increased FDG uptake in the right tongue base consistent with the lesion on CT scan that had SUV of 3.11.

He underwent endoscopy and biopsy in October 2011 and then was diagnosed with a tongue base recurrence by CT-guided core needle biopsy. His case was discussed by our institution’s Head and Neck tumor board and recommendation was made for repeat non-surgical treatment. Surgical resection is preferred at our institution for salvage of previously irradiated head and neck cancer. A variety of techniques are employed, including conventional open surgery, transoral laser surgery, and transoral robotic surgery. In his case, concern regarding compromise of speech and swallowing with surgery as well as his medical comorbidities prompted the recommendation of reirradiation.

**Figure 2:**
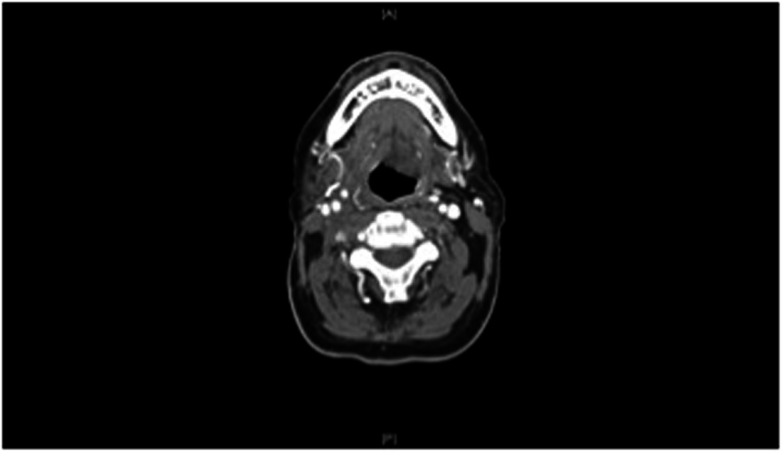
**After a 2-week course of stereotactic radiation, CT scan of the neck with contrast showing complete response to therapy**.

**Figure 3:**
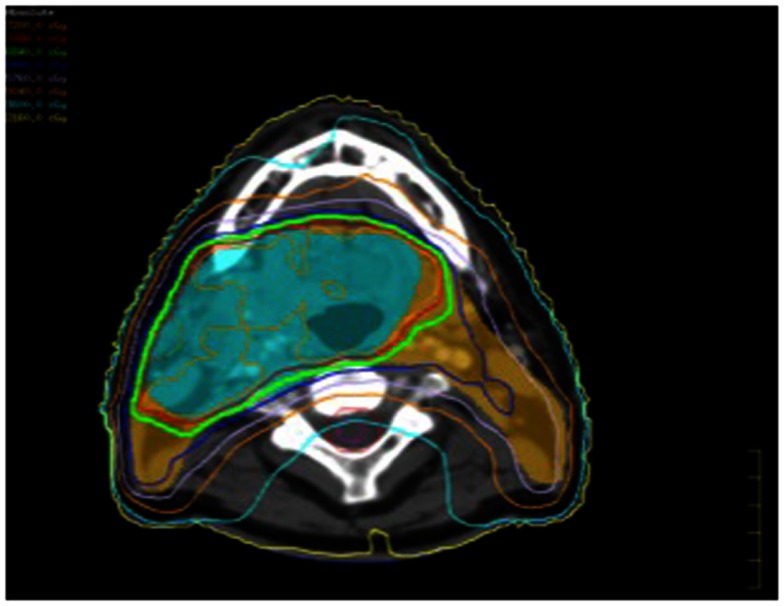
**The initial IMRT plan isodose lines (with the doses in the upper left corner)**.

**Figure 4:**
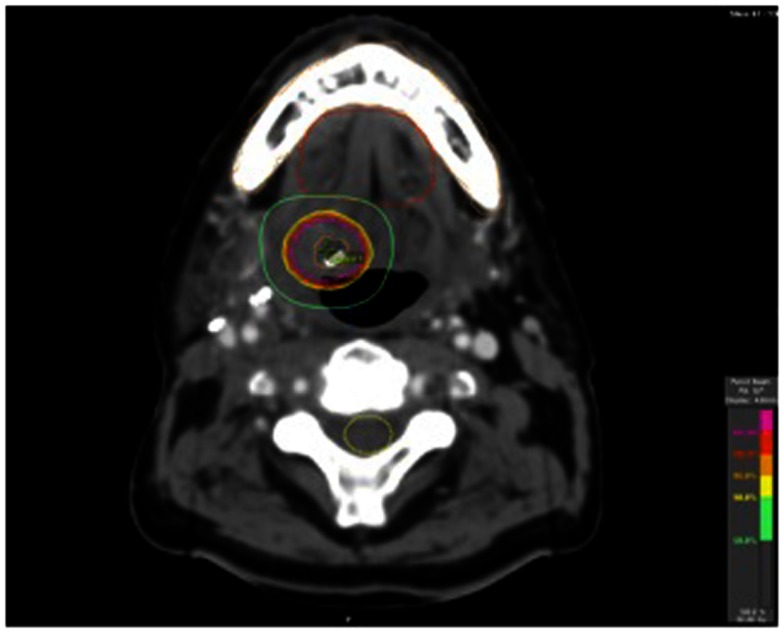
**The SRT plan isodose lines (with doses bottom right)**.

He subsequently had a fiducial placed in the tongue base mass and underwent a course of hypofractionated image-guided stereotactic radiation therapy (Figures 3 and 4). Daily cone beam CT was used to reduce setup variability. Fifty gray was delivered in 10 fractions of 5 Gy per fraction on a daily basis Monday through Friday. He tolerated this well. He returned to our clinic 3 months post treatment. Clinically he continued to do well, with mild xerostomia and chronic neck stiffness his only complaints. Comparison of his pre and post treatment CT scans showed clear and complete resolution of the submucosal tongue base mass (Figure 2). Likewise, pre and post treatment PET scans showed resolution of hypermetabolism in that location.

Two months later he did develop a small ulcer in the oropharynx at the posterior right tongue base and vallecula juncture at the SBRT treatment site. Given the proximity to therapy, this was suspected to be radiation induce mucositis and was treated conservatively as such. This improved and resolved with the aid of lidocaine containing mouthwash over a 2-month period. Repeat imaging 6 months post SBRT remained clear with no signs of disease recurrence.

## Discussion

The list of cancers amenable to treatment by SBRT is rapidly growing. It utilizes advanced radiation techniques to generate a steep dose gradient and allows for shortened treatment courses with high dose per fraction. Recently, SBRT has become a viable option for the treatment of recurrent head and neck cancer in previously irradiated patients who are not surgical candidates. Although there is currently limited data regarding its effectiveness in HPV positive patients, it can be expected to work well in this population of patients who is currently thought of as more responsive to traditional treatment.

There is debate about future direction of treatment of HPV positive head and neck cancer. As tobacco use declines, HPV positive cancers will become increasingly predominant. Because HPV positive cancers have good prognosis and better response to treatment compared to tobacco induced disease, current aggressive therapy may need to be compared to less aggressive therapy which might achieve similar disease outcome with less treatment related morbidity. As previously described, SBRT may fit the requirements for alternative therapy.

Early follow up of our patient shows encouraging results. With regard to HPV status, our institution uses a separate probe with a “cocktail” of different low- and high-risk types in addition to the commonly available subtype testing.

Although he was negative for commonly tested low- and high-risk HPV subtypes (6–11, 16–18, 31–33), positivity with our probe was confirmed indicating the presence of HPV DNA in the pathologic specimen. He has had a complete response to therapy with minimal toxicity. SBRT is shown in this case to be a possible alternative to surgical extirpation. Future studies should report on HPV status. It will be interesting to see the evolution of the role of SBRT in the treatment of HPV-related cancers.

## Conflict of Interest Statement

The authors declare that the research was conducted in the absence of any commercial or financial relationships that could be construed as a potential conflict of interest.
